# Direct comparisons of T cell costimulation and checkpoint blockade in the setting of tumor-targeted monoclonal antibody therapy

**DOI:** 10.1186/2051-1426-3-S2-P361

**Published:** 2015-11-04

**Authors:** Danny Khalil, Taha Merghoub, Jedd Wolchok

**Affiliations:** 1 Memorial Sloan Kettering Cancer Center, New York, NY, USA

## 

T cell checkpoint blockade and T cell costimulation, two types of immunotherapy undergoing active clinical investigation, have led to improved outcomes for patients with different types of advanced cancer. Given that these approaches can result in durable remissions, there is interest in increasing the number of patients who can benefit from them. Interestingly, there is evidence that patients who ultimately respond to T cell targeted immunotherapy are immunologically primed prior to treatment with an immune-activated tumor microenvironment. Importantly, recent data suggest that monoclonal antibodies (mAbs) against tumor antigens generate tumor-specific T cell activity. Our group has demonstrated that TA99, a murine IgG2a mAb against the melanosomal TYRP1 enzyme, can induce tumor-specific T cell responses; and others have shown that breast cancer patients treated with the humanized anti-HER2 IgG1 mAb trastuzumab also develop tumor-specific T cell responses. This raises the possibility that such antibodies may potentiate responses to immune checkpoint blockade and costimulation. While others have tested T cell stimulatory therapy in the setting of tumor targeted mAb treatment, we are not aware of a comparison of multiple T cell stimulatory mAbs in this context. We have therefore directly compared the ability of checkpoint blocking mAbs (against PD-1, PD-L1, and CTLA-4) and costimulatory mAbs (against OX40, 4-1BB, and GITR) to be potentiated by TA99. Using a syngeneic and poorly immunogenic murine melanoma model we have found enhanced tumor control with TA99 in combination with anti-CTLA-4 and anti-OX40 respectively. Crucially, TA99 in combination with these agents results in prolonged tumor-specific immunologic memory. This suggests that these regimens, and analogous immunotherapeutic couplets with other tumor-targeted mAbs, may yield durable anti-tumor activity in patients and should be explored in the clinic.

